# Consumo de carne vermelha e processada, resistência insulínica e diabetes no Estudo Longitudinal de Saúde do Adulto (ELSA-Brasil)

**DOI:** 10.26633/RPSP.2019.40

**Published:** 2019-05-03

**Authors:** Carla Moronari de Oliveira Aprelini, Vivian Cristine Luft, Gustavo Velásquez Meléndez, Maria Inês Schmidt, José Geraldo Mill, Maria del Carmen Bisi Molina

**Affiliations:** 1 Programa de Pós-Graduação em Saúde Coletiva Programa de Pós-Graduação em Saúde Coletiva Universidade Federal do Espírito Santo VitóriaES Brasil Universidade Federal do Espírito Santo, Programa de Pós-Graduação em Saúde Coletiva, Vitória (ES), Brasil.; 2 Programa de Pós-Graduação em Epidemiologia Programa de Pós-Graduação em Epidemiologia Universidade Federal do Rio Grande do Sul (UFRGS) Porto AlegreRS Brasil Universidade Federal do Rio Grande do Sul (UFRGS), Programa de Pós-Graduação em Epidemiologia, Porto Alegre (RS), Brasil.; 3 Escola de Enfermagem Escola de Enfermagem Universidade Federal de Minas Gerais (UFMG) Belo HorizonteMG Brasil Universidade Federal de Minas Gerais (UFMG), Escola de Enfermagem, Belo Horizonte (MG), Brasil.

**Keywords:** Carne vermelha, produtos da carne, resistência à insulina, diabetes mellitus, Brasil, Red meat, meat products, insulin resistance, diabetes mellitus, Brazil, Carne roja, productos de la carne, resistencia a la insulina, diabetes mellitus, Brasil

## Abstract

**Objetivo.:**

Investigar a associação entre consumo de carne vermelha e processada e a ocorrência de novos casos de resistência insulínica (RI) e diabetes *mellitus* (DM) em participantes do Estudo Longitudinal de Saúde do Adulto (ELSA-Brasil).

**Métodos.:**

Estudo de coorte com 15 105 servidores públicos (idade: 35 a 74 anos). Dados bioquímicos, antropométricos, socioeconômicos e de estilo de vida foram coletados na linha de base (2008–2010) e na segunda onda (2012–2014). O consumo de carnes (g/dia) foi estimado por questionário de frequência alimentar. Para categorizar baixo, médio e alto consumo as variáveis independentes foram divididas em tercis. DM foi diagnosticado como glicemia de jejum ≥ 126 mg/dL, glicose pós-sobrecarga ≥ 200 mg/dL ou hemoglobina glicada ≥ 6,5. RI foi determinada pelo índice HOMA-IR com pontos de corte construídos a partir do percentil 75 da amostra.

**Resultados.:**

Homens e participantes com menor renda e escolaridade relataram maior consumo de carne vermelha e processada. Maior consumo de carne processada (último tercil, > 27,1 g/dia) associou-se a novos casos de RI em homens (OR = 1,68; IC95%: 1,31 a 2,16) e mulheres (OR = 1,23; IC95%: 1,00 a 1,52). Alto consumo de carne vermelha aumentou em 40% (IC95%: 1,04 a 1,96) a chance de novos casos de DM em homens.

**Conclusões.:**

O consumo elevado de carne vermelha e processada teve impacto negativo na saúde dos participantes. O consumo moderado de carnes pode ser recomendado para a população em geral e para prevenção do DM.

## Abstract (en)

**Objective.:**

To investigate the association between red and processed meat consumption and the occurrence of new cases of insulin resistance (IR) and diabetes *mellitus* (DM) in participants of the Brazilian Longitudinal Study of Adult Health (ELSA-Brasil).

**Method.:**

This cohort study included 15 105 civil servants (age: 35-74 years). Biochemical, anthropometric, and socioeconomic data, as well as lifestyle characteristics, were collected at baseline (2008–2010) and wave 2 (2012–2014). Meat consumption (g/day) was estimated using a food frequency questionnaire. To categorize low, medium, and high consumption, independent variables were divided into tertiles. DM was diagnosed as fasting glucose ≥ 126 mg/dL, postload glucose ≥ 200 mg/dL, or glycated hemoglobin ≥ 6.5. IR was determined by HOMA-IR with cutoff points based on the sample’s 75th percentile.

**Results.:**

Men and participants with lower income and schooling reported higher consumption of red and processed meat. High consumption of processed meat (highest tertile, > 27.1 g/day) was associated with new cases of IR in men (OR = 1.68; 95%CI: 1.31-2.16) and women (OR = 1.23; 95%CI: 1.00-1>52). High consumption of red meat increased by 40% (95%CI: 1.04-1.96) the likelihood of new cases of DM in men.

**Conclusions.:**

High consumption of red/processed meat negatively impacted the health of participants. Moderate consumption of meats may be recommended for the general population and for prevention of DM.

## RESUMEN

**Objetivo.:**

Investigar la relación entre el consumo de carnes rojas y de carne procesada y la incidencia de nuevos casos de resistencia a la insulina y de diabetes mellitus en los participantes en el estudio longitudinal de salud del adulto (ELSA-Brasil).

**Métodos.:**

Estudio de cohorte con 15 105 funcionarios públicos (de 35 a 74 años de edad). Se recopilaron datos bioquímicos, antropométricos, socioeconómicos y sobre el estilo de vida en la línea de base (2008–2010) y en la segunda fase (2012–2014). Se calculó el consumo de carne (g/día) por medio de un cuestionario sobre la frecuencia del consumo de alimentos. Para las clasificaciones de consumo bajo, mediano y alto, las variables independientes se dividieron en terciles. La diabetes mellitus se diagnosticó como glucemia en ayunas ≥ 126 mg/dl, glucosa después de una sobrecarga ≥ 200 mg/dl o glucohemoglobina ≥ 6,5. La resistencia a la insulina se determinó con el modelo homeostático HOMA-IR con puntos de corte a partir del percentil 75 de la muestra.

**Resultados.:**

Los hombres y los participantes de menores ingresos y menor grado de escolaridad declararon un mayor consumo de carnes rojas y de carnes procesadas. El mayor consumo de carne procesada (último tercil, > 27,1 g/día) guardó relación con nuevos casos de resistencia a la insulina en los hombres (razón de probabilidades [OR] = 1,68; IC95%: 1,31-2,16) y las mujeres (OR = 1,23; IC95%: 1,00-1,52). El consumo alto de carnes rojas aumentó un 40% (IC95%: 1,04-1,96) la posibilidad de manifestación de nuevos casos de diabetes mellitus en los hombres.

**Conclusiones.:**

El consumo alto de carnes rojas y de carne procesada afectó desfavorablemente la salud de los participantes. El consumo moderado de carne puede recomendarse a la población en general y para la prevención de la diabetes mellitus.

De acordo com a Pesquisa de Orçamentos Familiares (POF) 2008-2009, a carne está entre os alimentos mais consumidos pelas famílias brasileiras, com contribuição de 12,6% ao valor calórico total. A POF mostrou também um aumento relativo de 15% no consumo de carne bovina e de 25% no consumo de embutidos ao longo de 6 anos (1). Conforme o Inquérito Nacional de Alimentação, o consumo de carne vermelha e carne processada no Brasil foi maior do que o recomendado pelo *World Cancer Research Fund, *de 43 g/dia de carne vermelha e quantidade mínima de carne processada, com mais 80% da amostra estudada consumindo acima da recomendação (2).

Existem evidências de associação entre alto consumo de carne vermelha e processada e ocorrência de diabetes *mellitus* (DM) e resistência insulínica (RI) em diferentes populações (3-6). O DM é um problema de saúde pública que acarreta aumento da morbimortalidade cardiovascular e redução da qualidade de vida (7); por sua vez, a RI é um fator preditor do DM tipo 2 (8), estando relacionada também às doenças cardiovasculares e à inflamação (9, 10).

Entretanto, a relação entre consumo de carne vermelha e processada e DM ainda é controversa. Micha et al. (11) identificaram, por meio de revisão sistemática, que apenas o consumo de carne processada está associado à incidência de DM. Da mesma forma, um estudo longitudinal também não encontrou associação entre carne vermelha e risco de DM (12). Sendo assim, até o momento, não foi possível identificar qual substância presente nesses alimentos está relacionada diretamente à ocorrência de DM (13).

No Brasil, um estudo multicêntrico de coorte cujo propósito é investigar a incidência e os fatores de risco para doenças crônicas, em particular as doenças cardiovasculares e o DM – o Estudo Longitudinal de Saúde do Adulto (ELSA-Brasil) (http://www.elsa.org.br/oelsabrasil.html) – diagnosticou o DM em 19% dos participantes na linha de base (14). Além disso, o ELSA-Brasil mostrou que o consumo moderado e alto de carne vermelha esteve associado a maior RI em homens, mas não em mulheres (15). A partir desses achados, o presente estudo teve como objetivo investigar a associação entre consumo de carne vermelha e processada e ocorrência de novos casos de RI e DM nos participantes do ELSA-Brasil.

## MATERIAIS E MÉTODOS

Trata-se de um estudo tipo coorte, realizado com dados das duas primeiras etapas do ELSA-Brasil (períodos de 2008 a 2010 e 2012 a 2014). O estudo multicêntrico ELSA-Brasil abrange 15 105 servidores ativos ou aposentados, de 35 a 74 anos, de universidades públicas ou instituição de pesquisa (Universidades Federais da Bahia, Espírito Santo, Minas Gerais e Rio Grande do Sul; Universidade de São Paulo; e Fundação Oswaldo Cruz).

### Participantes do estudo

O recrutamento e a seleção dos participantes do ELSA-Brasil foram descritos anteriormente (16). A linha de base foi executada de 2008 a 2010 e o seguimento (etapa 2), de 2012 a 2014. Para este estudo, foram construídos dois bancos de dados, com o objetivo de analisar RI e DM separadamente. Do banco 1 foram excluídos os casos prevalentes de RI, e do banco 2 foram excluídos os casos prevalentes de DM identificados na linha de base; de ambos os bancos foram excluídos participantes com histórico de eventos cardiovasculares (infarto agudo do miocárdio, insuficiência cardíaca congestiva e acidente vascular cerebral), câncer (exceto câncer de pele), participantes com índice de massa corporal (IMC) > 40 e participantes com valores implausíveis de consumo calórico (< 500 e > 6 000 kcal). Foram excluídos ainda os que não retornaram para a segunda etapa. Foram considerados perdas os participantes sem dados das exposições principais (consumo de carne vermelha e processada) ou do desfecho (desenvolvimento de DM e RI).

### Dados socioeconômicos e de vida

Informações socioeconômicas e demográficas e informações relativas aos hábitos de vida foram coletadas por meio de questionários estruturados. A investigação dos dados deu destaque aos determinantes sociais de saúde por meio de entrevistas presenciais (17).

### Medidas antropométricas

Peso, altura e circunferência da cintura (CC) foram coletados de forma padronizada, por meio de técnicas descritas por Lohman et al. (18, 19). O IMC foi calculado e utilizado para classificar o estado nutricional (20) conforme as seguintes categorias: baixo peso, IMC < 18,5; peso adequado, IMC ≥ 18,5 e < 24,9; sobrepeso, IMC ≥ 25 e < 29,9; e obesidade, IMC ≥ 30.

### Dados dietéticos

Para estimar consumo alimentar habitual foi utilizado o Questionário de Frequência Alimentar (QFA) do ELSA-Brasil (21), validado por Molina et al. (22). O QFA ELSA-Brasil é um questionário semiquantitativo com 114 itens que avalia o consumo habitual dos últimos 12 meses.

Foi calculado o consumo diário (g/dia) de carne vermelha, carne processada e carne vermelha total. Para carne vermelha foram considerados fígado/miúdos, bucho/dobradinha, carne de boi com osso (mocotó, costela, rabo), carne de boi sem osso (bife, carne moída, carne ensopada) e carne de porco. Para carne processada foram considerados linguiça/chouriço (salsichão), hambúrguer (bife), frios light (blanquet, peito de peru, peito de chester), presunto/mortadela/copa/salame/patê e bacon/toucinho/torresmo. A variável carne vermelha total foi calculada pela soma do consumo de carne vermelha e carne processada. A fim de categorizar o baixo, médio e alto consumo, foram criadas as variáveis carne vermelha, carne processada e carne total, divididas posteriormente em tercis de consumo. Para identificar associação entre nutrientes, independentemente do consumo energético total, as variáveis contínuas (carboidrato, proteína, gordura saturada, fibra e sódio) foram ajustadas por energia por meio do método residual (23).

### Variáveis clínicas

Foram coletadas amostras sanguíneas de acordo com o preconizado pela Sociedade Brasileira de Patologia Clínica/Medicina Laboratorial (24). Antes da coleta, os participantes foram informados sobre os procedimentos e verificou-se, por meio de questionário, se as orientações dadas haviam sido cumpridas. A coleta foi dividida em duas etapas: após jejum de 8 a 12 h e 2 h após ingestão de uma sobrecarga de glicose.

Para identificar indivíduos com DM previamente diagnosticado, os participantes responderam a seguinte pergunta: “você já foi informado anteriormente por um médico que você tem/teve diabetes (açúcar no sangue)?”. As opções de resposta eram “sim” ou “não”. Aqueles que não declararam diagnóstico prévio de DM tiveram seus valores laboratoriais avaliados e foram classificados como portadores de DM na presença de glicemia de jejum ≥ 126 mg/dL, glicose pós-sobrecarga ≥ 200 mg/dL ou ainda hemoglobina glicada (A1C) ≥ 6,5%.

Para determinar RI, utilizou-se o índice *Homeostasis Model Assessment for Insulin Resistance* (HOMA-IR), proposto por Matthews et al. (25), a partir da seguinte fórmula: insulina de jejum (mcU/mL) x glicose de jejum (mg/dL)/405 (6). Os pontos de corte foram construídos a partir da identificação do percentil 75 (P75) da amostra, sendo específicos para essa população. Foram considerados resistentes à insulina os participantes com valores HOMA-IR acima do P75 (4,3 para homens e 3,6 para mulheres). Apesar da ampla utilização do método matemático HOMA-IR para avaliação da RI, não há um consenso sobre valores adequados e/ou pontos de corte para a classificação de cada população (26).

### Análise estatística

Foram realizados o teste do qui-quadrado (χ^2^) e análise de variância (ANOVA) para testar diferenças entre variáveis categóricas e contínuas, respectivamente, e consumo total de carne vermelha. Para verificar associação entre consumo de carne vermelha e processada e RI e DM, foram construídos modelos de regressão logística binária bruto e ajustado, considerando variáveis da linha de base (idade, escolaridade, uso de tabaco, consumo de bebida alcoólica, atividade física, consumo de frutas, hortaliças e bebidas açucaradas, energia) e do seguimento (delta de IMC).

O processamento e a análise de dados foram realizados utilizando o software SPSS IBM Statistics versão 22.0. Um valor de *P* < 0,05 foi considerado como estatisticamente significativo para todos os testes.

### Aspectos éticos

O projeto ELSA-Brasil foi aprovado pelos comitês de ética em pesquisa de cada Centro de Investigação, sob os registros 669/06 (USP), 343/06 (FIOCRUZ), 041/06 (UFES), 186/06 (UFMG), 194/06 (UFRGS) e 027/06 (UFBA). Todos os participantes assinaram um termo de consentimento livre e esclarecido nas duas etapas, tendo sido garantido o anonimato das informações obtidas.

## RESULTADOS

Do total de 15 105 participantes, foram excluídos do primeiro banco: 3 647 casos prevalentes de RI, 611 participantes com histórico de eventos cardiovasculares, 347 com histórico de câncer, 53 com IMC > 40, 272 com valores implausíveis de consumo calórico, 671 que não participaram da etapa 2 e 37 participantes com dados faltantes das variáveis de exposição ou desfecho. Sendo assim, o primeiro banco incluiu 9 467 indivíduos, 55,7% do sexo feminino. No segundo banco, foram excluídos: 2 516 casos prevalentes de DM, 676 participantes com histórico de eventos cardiovasculares, 376 com histórico de câncer, 133 com IMC > 40, 284 com valores implausíveis de consumo calórico, 716 que não participaram da etapa 2 e 23 com dados faltantes, resultando em 10 381 participantes. Desses, 57,2% eram do sexo feminino (dados não mostrados em tabela).

As características gerais dos participantes do estudo, de acordo com o consumo total de carne, são apresentadas na tabela 1. Observaram-se resultados significativos (*P* < 0,001) em ambos os sexos para associação entre carne total e as variáveis socioeconômicas (escolaridade e renda) e idade. Indivíduos com consumo intermediário e alto de carne total apresentam menores médias de idade que os do 1º tercil de consumo. Da mesma maneira, a renda *per capita* foi menor entre indivíduos com alto consumo total de carne. O percentual de indivíduos com ensino superior completo ou pós-graduação foi maior na amostra de consumo mais baixo de carne total.

**TABELA 1:** Características socioeconômicas, estilo de vida e saúde segundo tercis de consumo total de carne por sexo, ELSA-Brasil, 2008 a 2010

		Consumo total de carne (g/dia)					
		Homens (n = 4 188)			Mulheres (n = 5 279)		
		0 a 68,4	68,5 a 128,1	128,2 a 677,9	0 a 51,7	51,8 a 97,2	97,3 a 752,7
Variável^a^ média ± DP ou n (%)		n = 1 396	n = 1 396	n = 1 396	n = 1 757	n = 1 762	n = 1 759
Idade (anos)^b^		51,9 ± 9,0^c^	50,3 ± 8,8^d^	49,9 ± 8,7^d^	52,4 ± 8,8^c^	50,1 ± 8,3^d^	50,0 ± 8,2^d^
Raça/cor branca (%)^e^		762 (55,5)	740 (53,8)	702 (50,7)	917 (52,9)	978 (56,0)	913 (52,1)
Escolaridade^b^							
	Fundamental incompleto	86 (6,2)	95 (6,8)	100 (7,2)	41 (2,3)	35 (2,0)	50 (2,8)
	Fundamental completo	66 (4,7)	99 (7,1)	139 (10,0)	80 (4,6)	55 (3,1)	85 (4,8)
	Médio completo	419 (30,0)	454 (32,5)	496 (35,5)	543 (30,9)	591 (33,5)	687 (39,1)
	Superior completo ou pós-graduação	825 (59,1)	748 (53,6)	661 (47,3)	1 093 (62,2)	1 081 (61,4)	937 (53,3)
Renda líquida *per capita*^b,f^		4,0 ± 3,2^c^	3,5 ± 2,9^d^	3,3 ± 2,7^g^	4,4 ± 3,5^c^	4,0 ± 3,1^d^	3,5 ± 2,8^g^
Tabagismo^b^							
	Nunca fumou (%)	839 (60,1)	752 (53,9)	691 (49,5)	1 183 (67,3)	1 119 (63,5)	1 043 (59,3)
Atividade física^h^							
	METs (minutos/semana)^i^	799,2 ± 1086,4	834,7 ± 1314,4	747,8 ± 1166,1	630,1 ± 1014,6^c^	566,7 ± 1050,4^c^	440,8 ± 841,8^d^
Circunferência da cintura (cm)^b^		90,8 ± 10,0^c^	91,8 ± 10,0^d^	93,1 ± 9,8^g^	83,3 ± 10,4^c^	84,1 ± 10,5^d^	85,1 ± 10,3^g^
Índice de massa corporal (kg/m^2^)^b^							
	< 24,9	662 (47,5)	618 (44,3)	525 (37,6)	936 (53,3)	857 (48,7)	771 (43,8)
	≥ 25 e < 29,9	603 (43,2)	631 (45,2)	698 (50,0)	599 (34,1)	648 (36,8)	691 (39,3)
	≥ 30 e ≤ 40	130 (9,3)	146 (10,5)	173 (12,4)	222 (12,6)	255 (14,5)	297 (16,9)
Álcool (g/d)^b^		7,1 ± 10,7^c^	9,9 ± 12,8^d^	12,9 ± 15,9^g^	2,7 ± 5,5^c^	3,5 ± 6,0^d^	4,0 ± 7,2^d^
Carne vermelha (g/d)^b^		29,2 ± 18,2^c^	75,1 ± 23,1^d^	171,0 ± 94,8^g^	20,1 ± 15,7^c^	53,0 ± 16,3^d^	129,1 ± 77,3^g^
Carne processada (g/d)^b^		10,7 ± 10,7^c^	22,6 ± 17,2^d^	42,8 ± 34,2^g^	7,5 ± 8,6^c^	18,8 ± 14,2^d^	31,1 ± 28,7^g^
Carne total (g/d)^b^		40,0 ± 20,5^c^	97,7 ± 17,0^d^	213,8 ± 91,9^g^	27,6 ± 16,1^c^	71,8 ± 13,2^d^	160,3 ± 78,8^g^
Frutas/hortaliças (g/d)^b^		991,7 ± 586,6^c^	976,8 ± 533,5^c^	1 093,6 ± 591,8^d^	965,8 ± 531,9^c^	911,1 ± 485,3^d^	1 019,0 ± 556,7^g^
Bebidas açucaradas (g/d)^b^		330,6 ± 331,0^c^	388,7 ± 337,2^d^	471,7 ± 393,4^g^	257,2 ± 283,9^c^	309,0 ± 300,1^d^	391,8 ± 359,9^g^
Energia (kcal/d)^b^		2 692,1 ± 886,1^c^	3 044,7 ± 891,1^d^	3 752,7 ± 987,1^g^	2 275,9 ± 760,0^c^	2 477,5 ± 745,3^d^	3 030,4 ± 963,6^g^
Proteína (g/d)^b,j^		122,3 ± 26,2^c^	125,3 ± 23,9^d^	135,3 ± 24,1^g^	127,2 ± 29,4^c^	131,1 ± 23,4^d^	137,8 ± 25,5^g^
Carboidrato (g/d)^b,j^		387,4 ± 54,4^c^	363,5 ± 48,7^d^	332,5 ± 52,3^g^	393,6 ± 57,3^c^	367,3 ± 50,0^d^	342,6 ± 53,9^g^
Ácido graxo saturado (g/d)^b,j^		26,2 ± 7,9^c^	28,7 ± 7,3^d^	31,8 ± 7,1^g^	27,6 ± 8,5^c^	30,9 ± 7,8^d^	33,6 ± 7,4^g^
Fibras (g/d)^b,j^		38,8 ± 12,1^c^	34,9 ± 9,9^d^	32,7 ± 10,2^g^	41,1 ± 12,1^c^	36,2 ± 10,3^d^	33,8 ± 10,0^g^
Sódio (mg/d)^b,j^		993,1 ± 166,7^c^	1 028,2 ± 142,4^d^	1 044,8 ± 141,1^g^	981,7 ± 176,0^c^	1 028,9 ± 157,6^d^	1 031,3 ± 156,0^d^

a Qui-quadrado e ANOVA para variáveis categóricas e contínuas, respectivamente.

b *P*-valor < 0,001 entre categorias de consumo dentro de cada sexo.

c,d,g Teste post-hoc de Tukey; letras iguais não diferem estatisticamente.

e *P*-valor < 0,05 para comparação entre categorias de consumo apenas nos homens.

f Salário mínimo em 2009 = US$ 202,18.

h *P*-valor < 0,05 para comparação entre categorias de consumo apenas nas mulheres.

i MET, equivalente metabólico por minuto por semana.

j Nutrientes ajustados por energia.

**FIGURA 1:**
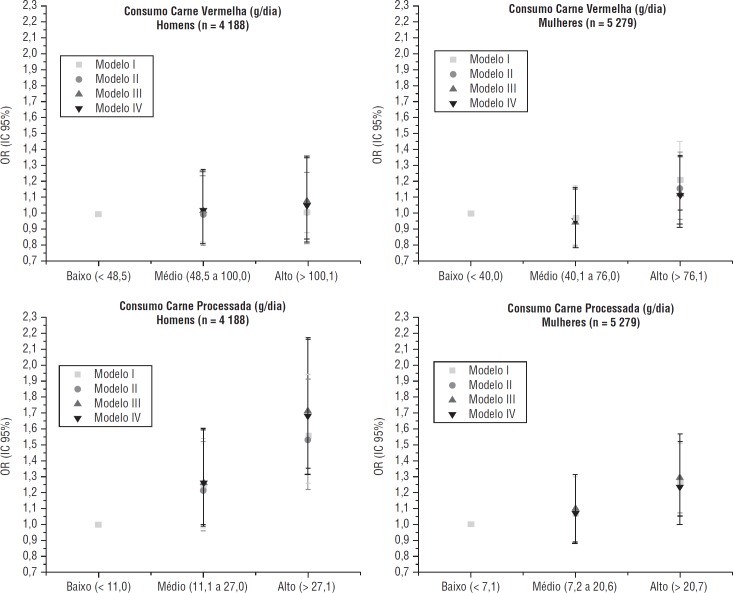
Consumo de carne e novos casos de HOMA-IR elevado por sexo, ELSA-Brasil, 2008 a 2010 e 2012 a 2014^a^

^a^ Regressão logística binária. OR significativo para *P*-valor < 0,05. Baixo consumo utilizado como referência. Modelo I: bruto; modelo II: ajustado por idade, escolaridade, tabagismo, consumo de álcool e atividade física; modelo III: modelo II + consumo de frutas/hortaliças, consumo de bebidas açucaradas e energia; modelo IV: modelo III + Δ IMC entre ondas (2008 a 2010 e 2012 a 2014). Resistência insulínica determinada pelo índice HOMA-IR com pontos de corte construídos a partir do percentil 75 (P75) da amostra.

Com relação aos hábitos de vida, foi observada associação significativa entre uso de tabaco e álcool e percentil de consumo de carne total (*P* < 0,001), em ambos os sexos. A maioria dos indivíduos que nunca havia fumado (60,1% e 67,3% em homens e mulheres, respectivamente) se enquadrava no tercil mais baixo de consumo total de carne, assim como os de menor consumo de álcool (g/dia). A atividade física apresentou valor significativo apenas no sexo feminino, com média menor de equivalente metabólico por minuto por semana (METs) nas mulheres com consumo mais elevado de carne total.

Variáveis antropométricas (CC e IMC) também apresentaram associação significativa com percentil de consumo de carne total. CC (em cm) foi maior nos indivíduos com maior consumo total de carne; ainda, houve maior porcentagem de participantes com IMC ≥ 30 (obesidade) nesse grupo. O consumo médio diário de carne vermelha, carne processada, bebidas açucaradas, energia, proteína, ácido graxo saturado (AGS) e sódio foi maior nos indivíduos do último tercil de consumo. De modo contrário, o consumo médio diário de carboidratos e fibras foi menor nos participantes com alto consumo de carne total. Ao analisar o consumo de carboidratos por ocorrência de RI e DM na onda 2, foi observado menor consumo entre os que apresentavam RI quando comparados aos que não apresentavam tal condição (352,9 ± 59,4 vs. 362,5 ± 55,9; *P* < 0,001). Nenhuma outra relação significativa foi observada (dados não apresentados em tabela).

**FIGURA 2:**
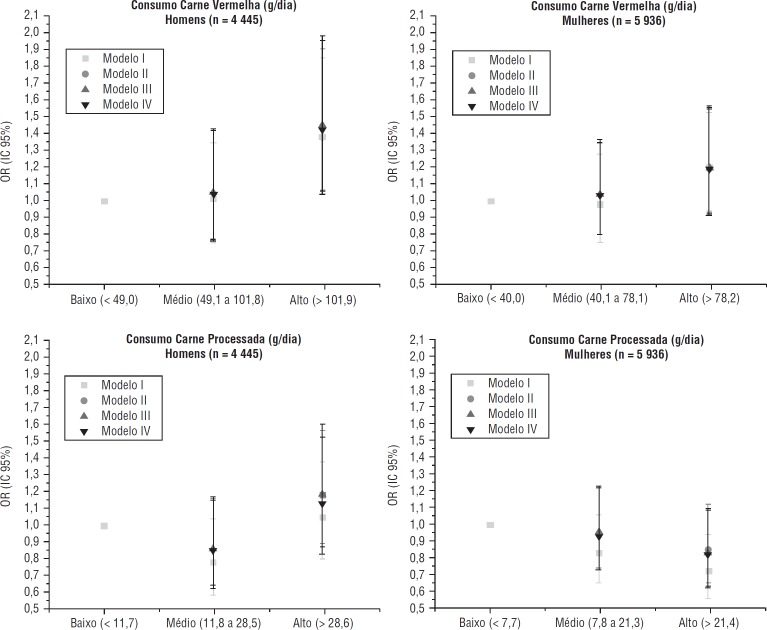
Consumo de carne e novos casos de diabetes por sexo, ELSA-Brasil, 2008 a 2010 e 2012 a 2014^a^

^a^ Regressão logística binária. OR significativo para *P*-valor < 0,05. Baixo consumo utilizado como referência. Modelo I: bruto; modelo II: ajustado por idade, escolaridade, tabagismo, consumo de álcool e atividade física; modelo III: modelo II + consumo de frutas/hortaliças, consumo de bebidas açucaradas e energia; modelo IV: modelo III + Δ IMC entre ondas (2008 a 2010 e 2012 a 2014).

**TABELA 2:** Consumo de carne e novos casos de HOMA-IR elevado por sexo, ELSA-Brasil, 2008 a 2010 e 2012 a 2014

A cada incremento de 100 g/dia^a^	Homens n = 4 188				Mulheres n = 5 279			
	Carne vermelha OR (IC95%)	*P*-valor^b^	Carne processada OR (IC95%)	*P*-valor^b^	Carne vermelha OR (IC95%)	*P*-valor^b^	Carne processada OR (IC 95%)	*P*-valor^b^
Modelo I	1,05 (0,95-1,16)	0,349	1,70 (1,26-2,30)	<0,001	1,14 (1,02-1,27)	0,017	1,43 (1,03-1,98)	0,033
Modelo II	1,00 (0,90-1,12)	0,957	1,62 (1,19-2,21)	0,002	1,09 (0,98-1,22)	0,123	1,46 (1,04-2,04)	0,027
Modelo III	1,04 (0,92-1,17)	0,545	1,89 (1,36-2,64)	<0,001	1,07 (0,95-1,21)	0,260	1,44 (1,00-2,06)	0,047
Modelo IV	1,00 (0,89-1,14)	0,881	1,73 (1,22-2,45)	0,002	1,05 (0,93-1,19)	0,412	1,19 (0,82-1,72)	0,359

a Modelo I: bruto; modelo II: ajustado por idade, escolaridade, tabagismo, consumo de álcool e atividade física; modelo III: modelo II + consumo de frutas/hortaliças, consumo de bebidas açucaradas e energia; modelo IV: modelo III + Δ IMC entre ondas (2008 a 2010 e 2012 a 2014). Resistência insulínica determinada pelo índice HOMA-IR com pontos de corte construídos a partir do percentil 75 (P&%) da amostra.

b Regressão logística binária. OR significativo para *P*-valor < 0,05.

**TABELA 3:** Consumo de carne e novos casos de diabetes por sexo, ELSA-Brasil, 2008 a 2010 e 2012 a 2014

A cada incremento de 100 g/dia^a^	Homens n = 4 445				Mulheres n = 5 936			
	Carne vermelha OR (IC95%)	*P*-valor^b^	Carne processada OR (IC95%)	*P*-valor^b^	Carne vermelha OR (IC95%)	*P*-valor^b^	Carne processada OR (IC 95%)	*P*-valor^b^
Modelo I	1,21 (1,08-1,36)	0,001	1,17 (0,77-1,78)	0,469	1,13 (0,98-1,31)	0,087	0,86 (0,53-1,41)	0,557
Modelo II	1,21 (1,07-1,37)	0,003	1,26 (0,81-1,94)	0,294	1,10 (0,95-1,28)	0,191	1,10 (0,68-1,78)	0,699
Modelo III	1,23 (1,07-1,41)	0,003	1,24 (0,78-1,98)	0,368	1,10 (0,93-1,30)	0,247	1,08 (0,65-1,81)	0,755
Modelo IV	1,22 (1,07-1,41)	0,004	1,13 (0,70-1,81)	0,618	1,09 (0,93-1,29)	0,294	1,07 (0,64-1,79)	0,807

a Modelo I: bruto; modelo II: ajustado por idade, escolaridade, tabagismo, consumo de álcool e atividade física; modelo III: modelo II + consumo de frutas/hortaliças, consumo de bebidas açucaradas e energia; modelo IV: modelo III + Δ IMC entre ondas (2008 a 2010 e 2012 a 2014).

b Regressão logística binária. OR significativo para *P*-valor < 0,05.

Na análise multivariada, incluindo possíveis variáveis de confusão, foi observada associação entre os tipos de carne e RI (figura 1). Em homens, foi encontrada associação positiva significativa entre alto consumo de carne processada e RI. Alto consumo de carne processada aumentou em 1,7 vez (*P *< 0,001; dado não mostrado) a chance de o indivíduo apresentar RI.

Em mulheres, o alto consumo de carne vermelha apresentou resultado significativo no modelo I. Contudo, esse resultado foi atenuado quando incluída a escolaridade. Novamente, o consumo alto de carne processada apresentou associação significativa (*P* < 0,05) com a ocorrência de RI, que se manteve mesmo com a inclusão de possíveis variáveis de confusão, mostrando que o consumo mais elevado de carne processada aumenta em 1,2 vez a chance de mulheres apresentarem resistência às ações da insulina.

A figura 2 mostra a regressão logística binária entre tipos de carne e novos casos de DM. Observa-se, entre os homens, que o alto consumo de carne vermelha apresentou associação significativa mesmo após inclusão de possíveis variáveis de confusão, aumentando em 1,4 vez a chance de o indivíduo apresentar DM. Em mulheres, não foi observada essa associação.

Na tabela 2 foi novamente identificada associação significativa entre consumo de carne processada e ocorrência de novos casos de RI em homens. A cada incremento de 100 g no consumo de carne processada por dia, aumentou em 1,7 vez a chance de o indivíduo apresentar RI. No sexo feminino, os valores foram atenuados com a inclusão da diferença de IMC entre as etapas 2 e 1 no último modelo. Não foi observada associação entre consumo de carne vermelha e RI nessa amostra.

Com relação ao consumo dos diferentes tipos de carne (exposição contínua e novos casos de DM) (tabela 3), foi encontrada associação positiva entre consumo de carne vermelha e ocorrência de DM apenas em homens, sendo que os valores se mantiveram significativos mesmo após inclusão de possíveis variáveis de confusão nos modelos. Isso indica que a cada incremento de 100 g no consumo diário de carne vermelha, aumentou em 1,2 vez a chance de os homens apresentarem DM.

## DISCUSSÃO

Os presentes resultados mostram que os participantes do ELSA-Brasil com maior consumo de carne total também apresentaram maior adiposidade abdominal, menor tempo semanal de atividade física e maior consumo de álcool. Há evidências de que esses fatores influenciam a ocorrência RI e DM (27-30). Desse modo, essas variáveis foram consideradas no modelo ajustado, bem como outras que também estiveram relacionadas com o desfecho e a exposição. Observou-se também menor ingestão média de carboidratos nos participantes com maior consumo total de carne; porém, o consumo de frutas e bebidas açucaradas foi maior nesses indivíduos. Portanto, o ajuste foi feito com essas duas últimas variáveis, as quais representam diretamente o consumo de carboidratos. Quando o consumo de carboidratos foi considerado na análise multivariada ao invés do consumo de frutas e açúcar, os resultados foram mantidos.

Assim sendo, detectou-se neste estudo uma associação significativa entre o alto consumo de carne processada e RI em homens, que se manteve mesmo após ajuste por variáveis de confusão. Um estudo recente de intervenção com 49 participantes identificou, entre os indivíduos com RI, que o padrão alimentar com alto consumo de carne vermelha e processada e grãos refinados diminuiu a sensibilidade à insulina quando comparado ao padrão dietético com alto consumo de grãos integrais, nozes, produtos lácteos e leguminosas (31). De modo contrário, uma pesquisa realizada com o objetivo de avaliar a relação entre consumo de carne vermelha total e incidência de síndrome metabólica e seus componentes em uma população do Mediterrâneo identificou que os indivíduos do último quartil de consumo de carne apresentaram maior risco para obesidade central quando comparados aos do quartil mais baixo, porém sem significância quanto à diminuição da tolerância à glicose (32). Nesse estudo, não houve associação significativa entre consumo de carne e sensibilidade à insulina, mas detectou-se maior risco para obesidade, que envolve um processo inflamatório crônico o qual se relaciona com a RI.

O presente estudo mostrou também uma associação significativa entre o consumo de carne processada e RI em mulheres. Ao realizar a análise multivariada com a exposição contínua, o valor foi atenuado com a inclusão da diferença de IMC das etapas 2 e 1 no último modelo. Outro estudo realizado na linha de base do ELSA-Brasil também encontrou associação significativa entre consumo de carne e RI apenas em homens (15).

No Canadá, um estudo concluiu que os homens, as pessoas de mais idade, os obesos e os sedentários apresentam maior risco para o desenvolvimento de RI (33). Por outro lado, Kautzky-Willer et al. (34), que realizaram estudo a fim de verificar o impacto do sexo no metabolismo da glicose, identificaram maior sensibilidade à insulina, verificada pelo método de QUICKI, nas mulheres do que nos homens, mesmo após considerar idade e IMC nas análises.

Alguns estudos desenvolvidos com mulheres não diabéticas com objetivo de analisar o consumo de carne vermelha (que incluía o consumo de carne processada) identificaram associação significativa entre alto consumo de carne, RI e homeostase anormal da glicose. O alto consumo de carne apresentou também relação com concentrações elevadas de biomarcadores da disfunção endotelial plasmática (5, 6).

A pré-diabetes está associada à disfunção de células β pancreáticas e resistência às ações da insulina. De acordo com Tabak et al. (35), mudanças nos hábitos de vida são primordiais para prevenir o DM (redução de 40-70% do risco) em indivíduos que já demonstram anormalidade na homeostase glicêmica.

Não foi constatado, no presente estudo, efeito entre o consumo dos tipos de carne e DM em mulheres, apenas nos homens. Souza et al. (36), ao caracterizarem o consumo alimentar da população brasileira com base no Inquérito Nacional de Alimentação 2008-2009, identificaram que os homens consomem mais carne bovina do que as mulheres, o que pode justificar os resultados encontrados.

Somente o consumo de carne vermelha, entre os homens, apresentou associação significativa com DM, mesmo com a inclusão de possíveis variáveis de confusão. Estudos de metanálise associaram de forma significativa o consumo tanto de carne vermelha quanto de carne processada e o risco elevado de desenvolver DM (3, 4). Do mesmo modo, um estudo de caso-controle aninhado à coorte (37) também identificou um risco aumentado para DM nos indivíduos com maior consumo de carne vermelha e carne processada. Barnard et al. (38) sugerem, em seu estudo de revisão, que o consumo de carne vermelha está consistentemente associado ao risco de DM.

Männistö et al. (12) não relataram associação positiva entre o consumo de carne vermelha e DM do tipo 2. Entretanto, encontraram relação de risco quando analisaram os quintis de consumo de carne vermelha total (RR = 1,50; IC95%: 1,23 a 1,82) e de carne processada (RR = 1,37; IC95%: 1,11 a 1,71). Lenighan et al. (39), em estudo transversal, não encontraram associação positiva entre biomarcadores de doenças cardiovasculares e DM tipo 2 e o padrão dietético com contribuição elevada de carne vermelha e processada.

Elementos presentes na carne vermelha e processada podem estar associados à ocorrência de RI e DM – entre eles, maior quantidade de ácidos graxos saturados, maior concentração de sódio, produtos finais de glicação avançada (AGEs) formados no processo de cozimento da carne e formação de nitrosaminas durante o processamento de carne, dentre outros (13). Outras investigações devem ser realizadas a fim de identificar a via que relaciona o efeito do alto consumo de carne à ocorrência de DM e RI.

O presente estudo apresenta limitações, entre as quais a utilização do QFA. Como outros métodos dietéticos, o QFA depende da memória dos participantes, da compreensão por parte do participante do que o pesquisador deseja mensurar e do tempo de coleta (40). Apesar disso, o QFA é o método indicado em estudos epidemiológicos para investigar associações entre nutrientes/alimentos e desfechos e apresenta custo reduzido quando comparado a outros instrumentos de coleta de dados. Além disso, houve enorme esforço durante o planejamento do estudo para minimizar tais problemas, com treinamento centralizado, certificação e recertificação após 6 meses. Outra medida adotada para o controle de qualidade foi a utilização, durante a coleta de dados, de cartões de resposta e kit padronizado de utensílios para aferição em medidas caseiras; ademais, foi realizado estudo para avaliar a reprodutibilidade e validade do QFA ELSA-Brasil (17, 22). Vale ressaltar que foi feito o ajuste de energia para os nutrientes estudados por meio do método residual.

O atual estudo utilizou duas etapas (ondas) de coleta de dados, mas não foi possível identificar quando ocorreram os novos casos de RI/DM. Assim, selecionamos os indivíduos com esses agravos na segunda etapa, o que impossibilitou a contagem das pessoas-tempo e consequentemente as análises de risco relativo. Entretanto, nosso estudo, que é uma coorte multicêntrica com amostra grande e prospectiva, minimiza ou anula a causalidade reversa, além de permitir estimar o risco dos agravos analisados.

O tempo de observação entre ondas foi pequeno, não sendo um período suficiente para o surgimento de novos casos de DM. É de fundamental importância a continuidade do acompanhamento para determinação de novos casos de DM, visto que o fator idade é de grande relevância para a ocorrência da doença. Entretanto, foi possível identificar o efeito do consumo da carne vermelha e carne processada na RI.

Devido ao grande porte do estudo ELSA-Brasil e à sua natureza multicêntrica, utilizaram-se vários mecanismos para assegurar a qualidade do estudo. Entre eles, merecem destaque as atividades desenvolvidas antes e durante a coleta de dados e durante o processamento de dados, como treinamento, certificação e recertificação dos pesquisadores, escolha criteriosa dos instrumentos de coleta, validação de questionários, estudo piloto e monitoramento dos dados, dentre outros (41).

Levando-se em consideração o aumento dos casos de DM, a gravidade dessa doença e suas complicações e associação com doenças cardiovasculares, é de suma importância a identificação de fatores de risco e tratamento precoce dessa enfermidade. Sendo o DM um problema de saúde pública que leva à perda de qualidade de vida dos acometidos e gera alto custo para o sistema de saúde, são necessárias ações de prevenção, dentre as quais está a mudança no hábito de consumir carne vermelha e processada.

Em conclusão, o presente estudo mostrou que o consumo elevado de carne vermelha e processada foi associado a novos casos de DM e RI em homens. Em mulheres, apenas o alto consumo de carne processada aumentou a chance de ocorrência de novos casos de RI. Levando-se em consideração que a RI é um preditor independente para o DM, e este um fator de risco para as doenças cardiovasculares, é importante recomendar, em guias alimentares e nas estratégias de prevenção do DM, o consumo moderado desses componentes dietéticos.

## Contribuição dos autores.

CMOA realizou análises, interpretação dos dados e redação do artigo. VCL contribuiu com análise dos dados e revisão crítica do conteúdo. JGM, MIS, GVM contribuíram para o desenho e aquisição de dados. MCBM contribuiu para o desenho e análise de dados e revisou criticamente o conteúdo. Todos os autores aprovaram a versão final.

## Agradecimentos.

Os autores agradecem aos participantes do ELSA-Brasil.

## Financiamento.

Conselho Nacional de Desenvolvimento Científico e Tecnológico (CNPq), Coordenação de Aperfeiçoamento de Pessoal de Nível Superior (CAPES), Ministério da Ciência e Tecnologia – Brasil – Governo Federal.

## Declaração.

As opiniões expressas no manuscrito são de responsabilidade exclusiva dos autores e não refletem necessariamente a opinião ou política da RPSP/PAJPH ou da Organização Pan-Americana da Saúde (OPAS).
